# Supplementary material for: Determining hexavalent chromium transport properties in alkaline nuclear waste using nuclear magnetic resonance spectroscopy
Source: Commun Chem. 2025 Jun 7;8:180. doi: 10.1038/s42004-025-01546-7 (PMC12145419; doi:10.1038/s42004-025-01546-7)
Supplement: Supplementary file 2 — Supporting Information [file 42004_2025_1546_MOESM2_ESM.pdf]

# SUPPLEMENTARY INFORMATION

## Determining Hexavalent Chromium Transport Properties in Alkaline Nuclear Waste Using Nuclear Magnetic Resonance Spectroscopy

Trent R. Graham,[A,*] Ashley R. Kennedy,[A,B] Jacob Morton,[A] Jacob G. Reynolds,[C] and Carolyn I. Pearce[A,D]

[A] Pacific Northwest National Laboratory, Richland, Washington 99354, USA
[B] Savannah River National Laboratory, Aiken, South Carolina 29808, USA
[C] Central Plateau Cleanup Company, Richland, Washington 99354, USA
[D] Department of Crop and Soil Sciences, Washington State University, Pullman, Washington 99164, USA

## S1. SUPPLEMENTARY METHODS

Interpolation of literature dynamic viscosity coefficients for temperatures of 20, 40 and 60 °C and extrapolation of literature coefficients to estimate the viscosity at 80 °C was performed to estimate the dynamic viscosity of 1.75 m $K_2CrO_4$ in 1.47 m KOH, using the data of Guo et al.[1] Guo et al. measured the density and viscosity of a series of KOH-$K_2CrO_4$ solutions using densimeters and Ubbelohde-type capillary viscometers, respectively. The compositions of their solutions were given in units of molarity (M), which is defined by moles/L. Given that the density of the solutions changes as a function of temperature, the solution prepared in this work was defined in terms of molality (m), which is defined by moles/$kg_{solvent}$. In this case, the solvent is water.

To relate molarity and molality, the mass of water in aqueous solution for each system bracketing the concentration used in this study were calculated using **Equation S1**

$$w_{H_2O} = \rho - \sum_i C_i \cdot MW_i \qquad \textbf{Equation S1}$$

where $w_{H_2O}$ is the weight of water in 1 mL of solution, ρ is the density of solution at 15°C in grams/mL, $C_i$ is the concentration of species $i$ in moles/L, $MW_i$ is the molecular weight of species $i$ in grams/mole. There are two species, KOH and $K_2CrO_4$. The molecular weight of KOH is 56.106 grams/mole and the molecular weight of $K_2CrO_4$ is 194.1896 grams/mole. Note that densities between 15 and 25 °C varied by less than 1%, which was assumed to be negligible.

Next, molarity of the solutions of Guo et al. were converted to molality using **Equation S2**.

$$m_i = \frac{C_i}{w_{H_2O}} \qquad \textbf{Equation S2}$$

Given **Equation S1** and **S2**, the concentrations of KOH-$K_2CrO_4$ solutions bracketing the KOH-$K_2CrO_4$ solution used in this study are listed in **Table S1** and illustrated in **Figure S1**.

**Table S1.** Compositions bracketing the solution composition used in this study.

| System Number | $C_{KOH}$ (M) | $C_{K_2CrO_4}$ (M) | $w_{H_2O}$ (g/mL) | *ρ (g/mL) | $m_{KOH}$ (m) | $m_{K_2CrO_4}$ (m) |
|---|---|---|---|---|---|---|
| 1 – Guo et al. | 1.00 | 1.07 | 0.93 | 1.20 | 1.07 | 1.15 |
| 2 – Guo et al. | 1.00 | 1.66 | 0.88 | 1.26 | 1.13 | 1.88 |
| 3 – Guo et al. | 1.01 | 2.11 | 0.85 | 1.31 | 1.20 | 2.49 |
| 4 – Guo et al. | 1.5 | 1.02 | 0.93 | 1.21 | 1.62 | 1.10 |
| 5 – Guo et al. | 1.51 | 1.79 | 0.89 | 1.32 | 1.69 | 2.01 |
| 6 – Guo et al. | 1.51 | 1.59 | 0.89 | 1.28 | 1.70 | 1.80 |
| 7 – This study | ** | ** | ** | **1.27 | 1.47 | 1.75 |

(*) Densities (ρ) reported at 15 °C. The density variation between 15°C and 25°C was less than 1%. This change is negligible when the density is rounded to two decimal places.
(**) Interpolated estimate.

For each data row, density and viscosity values were determined at various temperatures (15°C, 25°C, 30°C, 40°C, 50°C, 60°C). For the 20 °C interpolation, given values at 15°C and 25°C, Equation S3 was used:

$$P(20°) = P(15°C) + \frac{5}{10}\big(P(25°C) - P(15°C)\big) \quad \textbf{Equation S3}$$

These steps were repeated for density and viscosity across all data rows. Seven known data points $\big((m_{\text{KOH,i}}), (m_{\text{K}_2\text{CrO}_4\text{,i}})\big)$, with properties at all temperatures to interpolate the target point:

$$m_{\text{KOH,target}} = 1.47387, \quad m_{\text{K}_2\text{CrO}_4\text{,target}} = 1.751007$$

For each known point (i):

$$d_i = \sqrt{\left(m_{\text{KOH},i} - m_{\text{KOH,target}}\right)^2 + \left(m_{\text{K}_2\text{CrO}_4,i} - m_{\text{K}_2\text{CrO}_4\text{,target}}\right)^2} \quad \textbf{Equation S4}$$

where the normalized weights were calculated, let $w_i' = 1/d_i$:

$$w_i = \frac{w_i'}{\sum_j w_j'} \quad \textbf{Equation S5}$$

To extrapolate to viscosities at 80°C the data between 15 and 60°C was fit to an Arrhenius equation which was then used to extrapolate.

**Table S2.** Viscosities and compositions bracketing the solution composition used in this study.

| | | | Dynamic Viscosity (cp) | | | | | | | |
|---|---|---|---|---|---|---|---|---|---|---|
| System Number | $m_{\text{KOH}}$ (m) | $m_{\text{K}_2\text{CrO}_4}$ (m) | 15 °C | 20 °C | 25 °C | 30 °C | 40 °C | 50 °C | 60 °C | 80 °C |
| 1 – Guo et al. | 1.07 | 1.15 | 1.6 | N/A | 1.3 | 1.2 | 1 | 0.8 | 0.7 | N/A |
| 2 – Guo et al. | 1.13 | 1.88 | 1.7 | N/A | 1.4 | 1.3 | 1.1 | 0.9 | 0.8 | N/A |
| 3 – Guo et al. | 1.2 | 2.49 | 1.9 | N/A | 1.6 | 1.4 | 1.2 | 1 | 0.9 | N/A |
| 4 – Guo et al. | 1.62 | 1.1 | 1.6 | N/A | 1.3 | 1.2 | 1 | 0.9 | 0.7 | N/A |
| 5 – Guo et al. | 1.69 | 2.01 | 2.1 | N/A | 1.7 | 1.5 | 1.3 | 1.1 | 0.9 | N/A |
| 6 – Guo et al. | 1.7 | 1.8 | 1.9 | N/A | 1.5 | 1.4 | 1.2 | 1 | 0.9 | N/A |
| 7 – This study | 1.47 | 1.75 | 1.9 | 1.7 | 1.5 | 1.4 | 1.2 | 1 | 0.9 | 0.6 |

S2. SUPPLEMENTARY TABLES

**Table S3.** Compositions of Tank Waste Simulants[a]

| Analyte | S1 (mM) | S2 (mM) | S3 (mM) | S4 (mM) | S5 (mM) | Water[b] |
|---|---|---|---|---|---|---|
| $CrO_4^{2-}$ | 3.58 | 4.75 | 1.94 | 4.3 | 16.5 | 0 |
| $Al(OH)_4^-$ | 164 | 47.2 | 25.8 | 73 | 73.9 | 0 |
| $C_2O_4^{2-}$ | 9.77 | 15.6 | 14.2 | 19 | 20.2 | 0 |
| $Ca^{2+}$ | 0.737 | 0.631 | 0.658 | 1.99 | 0.205 | 0 |
| $Cl^-$ | 111 | 29.9 | 23.4 | 42.9 | 42.5 | 0 |
| $CO_3^{2-}$ | 427 | 171 | 129 | 208 | 230 | 0 |
| $Cs^+$ | 0.0281 | 0.00598 | 0.00519 | 0.00909 | 0.00877 | 0 |
| $F^-$ | 11.5 | 10 | 21.9 | 18.3 | 21.7 | 0 |
| $K^+$ | 36.5 | 9.7 | 6.68 | 14.6 | 9.78 | 0 |
| $Na^+$ | 5730 | 5030 | 1860 | 3450 | 4040 | 0 |
| $NO_2^-$ | 1110 | 250 | 228 | 421 | 370 | 0 |
| $NO_3^-$ | 1590 | 3640 | 849 | 1570 | 2130 | 0 |
| $OH^-$ | 1400 | 473 | 314 | 813 | 793 | 0 |
| $PO_4^{3-}$ | 43.3 | 48.4 | 44.2 | 69.5 | 43.6 | 0 |
| $SO_4^{2-}$ | 39.6 | 37.5 | 21.6 | 44.9 | 48.2 | 0 |
| $Sr^{2+}$ | 0.0033 | 0.00362 | 0.00339 | 0.00342 | 0.00777 | 0 |
| $CH_3CO_2^-$ | 30.8 | 1.3 | 11.4 | 10.8 | 0 | 0 |
| $HCO_2^-$ | 30.8 | 1.3 | 11.4 | 10.8 | 0 | 0 |
| $^{53}Cr$ NMR Integral[c] | 10.7 | 16.1 | 8.0 | 16.0 | 60.4 | -0.7 |

a. Schonewell et al., 2024[3]
b. Deionized water was used as a blank sample with an assumed concentration of 0 mM for all analytes.
c. The results of this work, in arbitrary units of counts.

**Table S4. S** $^{53}Cr$ NMR $T_1$ coefficients for 1.75 m $K_2CrO_4$ in 1.47 m KOH

| Temp (°C) | $T_1$ (ms) | +σ (ms) | -σ (ms) |
|---|---|---|---|
| 20 | 60.0 | 16.0 | 11.0 |
| 40 | 68.6 | 19.3 | 13.3 |
| 60 | 77.7 | 11.7 | 11.2 |
| 80 | 82.6 | 22.0 | 15.2 |

**Table S5.** $^{53}Cr$ NMR $T_2$ coefficients for 1.75 m $K_2CrO_4$ in 1.47 m KOH

| Temp (°C) | $T_2$ (ms) | +σ (ms) | -σ (ms) |
|---|---|---|---|
| 20 | 35.8 | 5.7 | 8.2 |
| 40 | 39.6 | 6.7 | 9.3 |
| 60 | 48.7 | 7.7 | 11.1 |
| 80 | 53.8 | 6.1 | 6.0 |

**Table S6.** $^{53}$Cr NMR $\tau_c$ coefficients for 1.75 m $K_2CrO_4$ in 1.47 m KOH

| Temp (°C) | $\tau_c$ (ms) | +σ (ms) | -σ (ms) |
|---|---|---|---|
| 20 | 4.8 | 2.4 | 1.7 |
| 40 | 5.2 | 3.2 | 2.1 |
| 60 | 4.6 | 2.5 | 1.7 |
| 80 | 4.2 | 2.8 | 1.9 |

**Table S7.** $^{53}$Cr NMR $D_T$ coefficients for 1.75 m $K_2CrO_4$ in 1.47 m KOH

| Temp (°C) | $D_T$ (ms) | +σ (ms) | -σ (ms) | Notes |
|---|---|---|---|---|
| 20 | 10.0 | 1.9 | 1.8 | PFGSTE NMR |
| 20 | 9.4 | 1.8 | 1.2 | Relaxometry |
| 40 | 12.0 | 2.4 | 1.6 | Relaxometry |
| 60 | 15.4 | 2.6 | 1.9 | Relaxometry |
| 80 | 19.4 | 3.9 | 2.8 | Relaxometry |

S3. SUPPLEMENTAL FIGURES

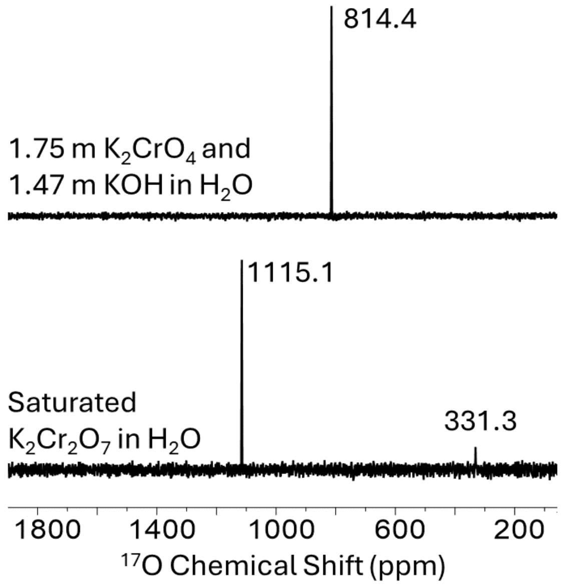


**Figure S1**. Single pulse direct excitation $^{17}$O NMR spectra of the $K_2CrO_4$ in KOH solution and a reference solution comprised of saturated $K_2Cr_2O_7$ in $H_2O$. The terminal Cr-O (1151.1 ppm) and bridging Cr-O-Cr (331.3 ppm) are observable in the reference solution and absent in the $CrO_4^{2-}$ (814.4 ppm) solution, confirming that the concentration of KOH was sufficient to speciate Cr(VI) into $CrO_4^{2-}$. The assignments are based on the work of others.[2] Single-pulse, direct excitation $^{17}$O NMR spectra were collected at field strength of 11.76 T, a temperature of 20 °C, with a time domain size of 3947 points, a sweep width of 200k Hz, and an acquisition time of 0.0197 s. A $\pi/20$ pulse width corresponding with 4 μs was applied. The spectra are referenced to deionized water (0 ppm). The relaxation delay was set to 0.4 s, and 32768 scans were performed. The spectra were analyzed in Mestrenova, zero-filled to 65536 complex points and a 20 Hz exponential window function was applied. The spectra are vertically normalized to the maximum peak height in the displayed region.

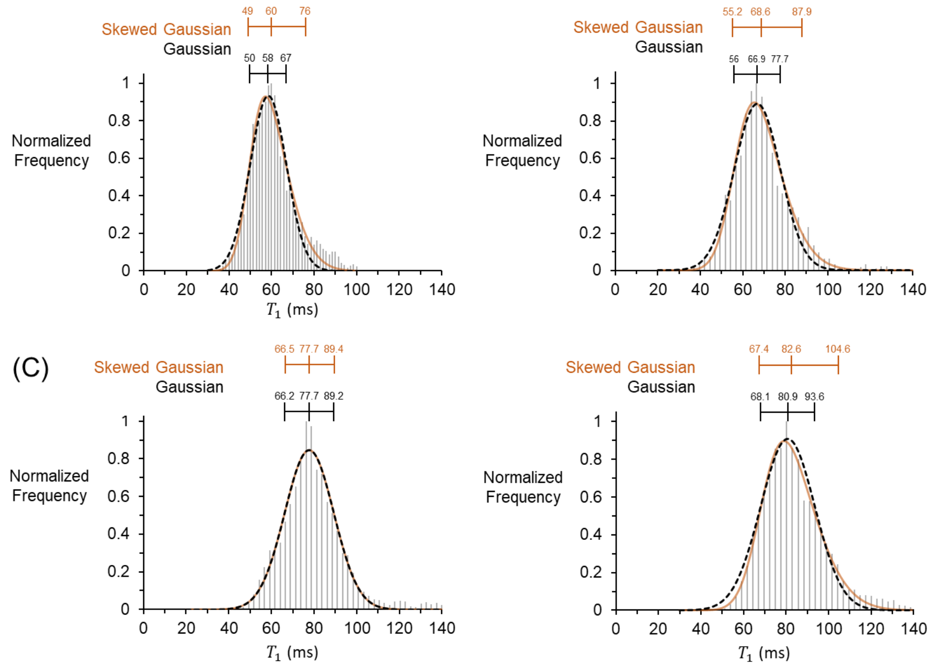


**Figure S2.** Monte Carlo simulations of saturation recovery $^{53}Cr$ NMR experiments at (A) 20 °C, (B) 40 °C (C) 60 °C and (D) 80 °C. The center of mass as well as 16% and 84% values are annotated for the skewed Gaussian and the Gaussian fits to the distribution of $T_1$. Uncertainties (+/- σ) are annotated.

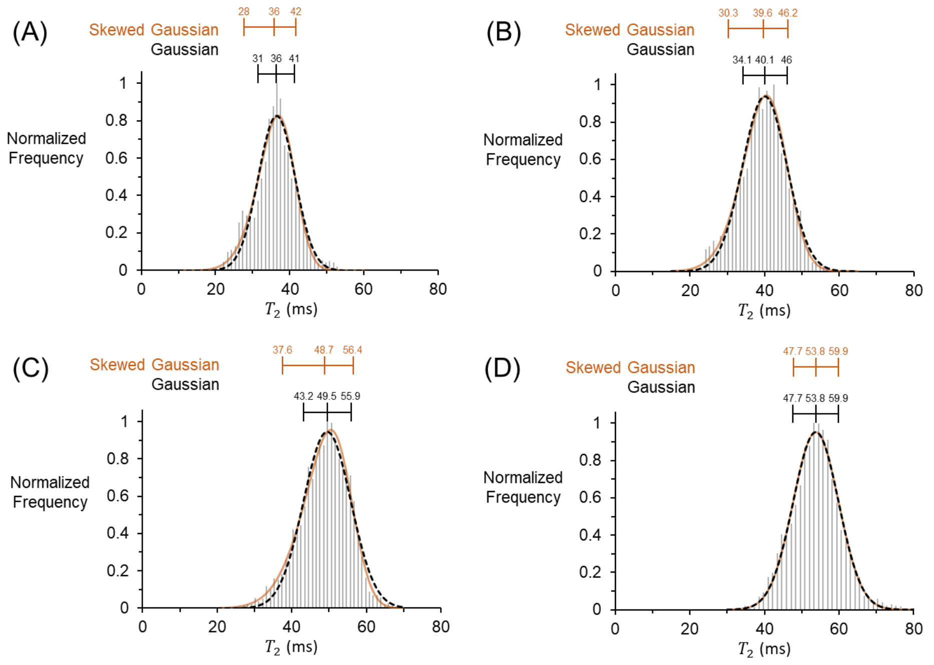


**Figure S3.** Monte Carlo simulations of $^{53}Cr$ CPMG NMR experiments at (A) 20 °C, (B) 40 °C (C) 60 °C and (D) 80 °C. The center of mass as well as 16% and 84% values are annotated for the skewed Gaussian and the Gaussian fits to the distribution of $T_2$. Uncertainties (+/- σ) are annotated.

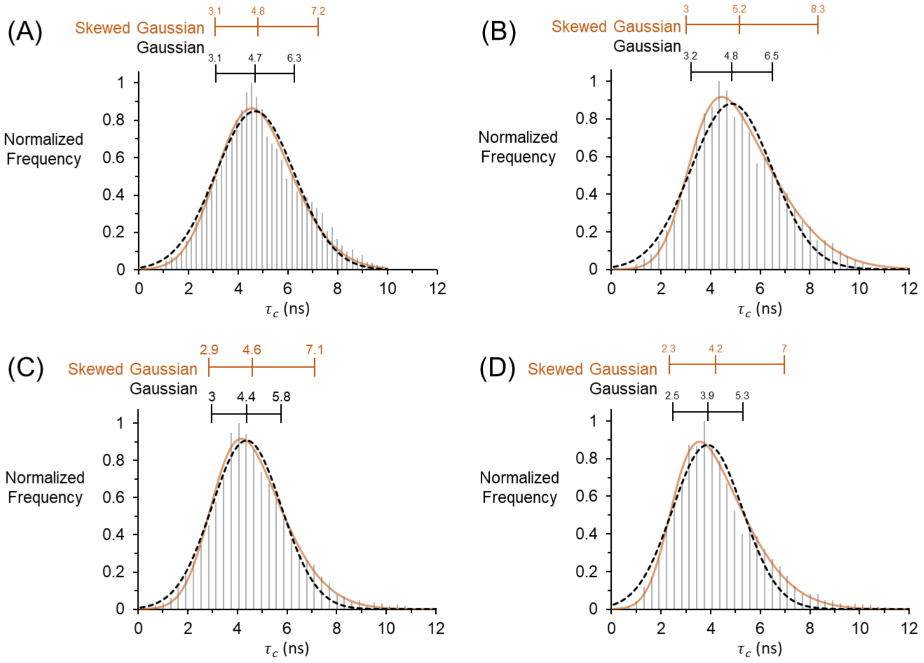


**Figure S4.** Monte Carlo simulations of rotational correlation coefficients at (A) 20 °C, (B) 40 °C (C) 60 °C and (D) 80 °C. The center of mass as well as 16% and 84% values are annotated for the skewed Gaussian and the Gaussian fits to the distribution of $\tau_c$. Uncertainties (+/- σ) are annotated.

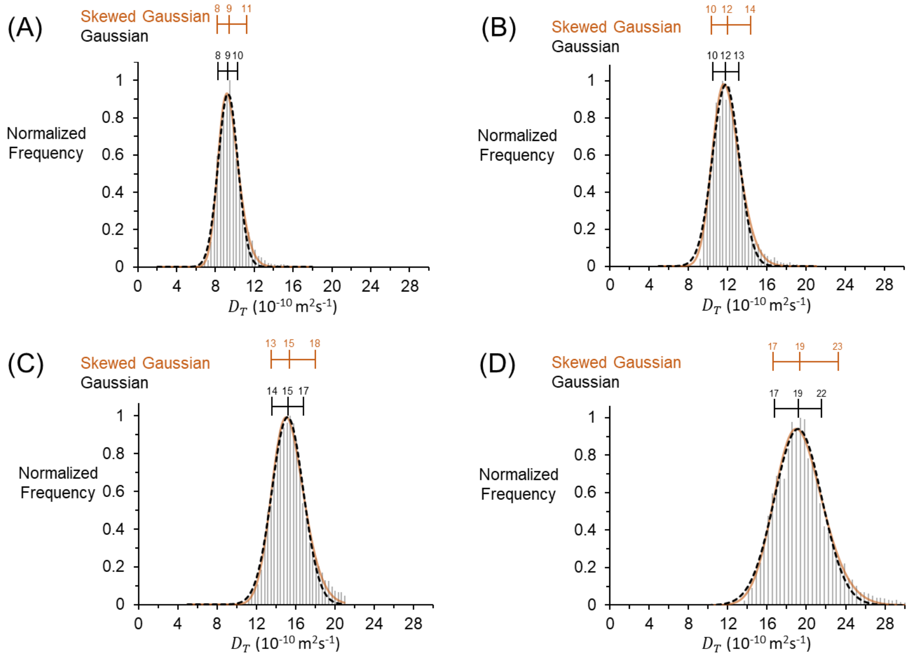


**Figure S5.** Monte Carlo simulations of $^{53}$Cr NMR relaxometry experiments at (A) 20 °C, (B) 40 °C (C) 60 °C and (D) 80 °C. The center of mass as well as 16% and 84% values are annotated for the skewed Gaussian and the Gaussian fits to the distribution of $T_1$. Uncertainties (+/- σ) are annotated.

S4. REFERENCES


1. Y.-j. Guo, H.-b. Xu, F. Guo, S.-l. Zheng and Y. Zhang, *Transactions of Nonferrous Metals Society of China*, 2010, **20**, s32-s36.

2. N. E. Brasch, D. A. Buckingham, A. B. Evans and C. R. Clark, *J Am Chem Soc*, 1996, **118**, 7969-7980.

3. Schonewell, P. E. K., LG; Burns. CA; Daniel, RA. Simulant Development of Potential 200 West Area Waste Feeds. (Pacific Northwest National Laboratory, 2024)
